# AP1 Transcription Factor in the Regulation of the Urokinase Plasminogen Activation System

**DOI:** 10.3390/biom16060778

**Published:** 2026-05-26

**Authors:** Petra Korać, Mariastefania Antica, Maja Matulić

**Affiliations:** Faculty of Science, University of Zagreb, Horvatovac 102, 10000 Zagreb, Croatia; petra.korac@biol.pmf.hr (P.K.); antica216@outlook.com (M.A.)

**Keywords:** urokinase plasminogen activator, PAI1, urokinase plasminogen activator receptor, AP1, promoter

## Abstract

Urokinase plasminogen activation system regulates the activation of plasminogen to produce the ubiquitous extracellular protease plasmin. It is involved in different physiological and pathophysiological processes, which involve tissue reorganization, wound healing, cell migration and invasion, etc. The system comprises urokinase plasminogen activator, an extracellular protease, its inhibitor plasminogen activator inhibitor PAI1 and urokinase receptor, uPAR. The system is regulated at the level of transcription and posttranscriptionally, and the net urokinase activity depends on the balance between urokinase and PAI1. Promoters of *urokinase*, *PAI1* and *uPAR* are regulated through different signaling pathways, mostly MAP kinases and TGFβ signaling. *Urokinase* promoter is complex and mostly depends on strong enhancers containing AP1/ETS binding sites for different combinations of AP1 dimers, whose members are phosphorylated through ERK, JNK and p38 kinases. The *PAI1* promoter is mainly regulated through TGFβ signaling, which can use both Smad and AP1-dependent transcription. The *uPAR* promoter also depends on AP1 signaling, in addition to other transcription factors activated through other pathways. Although activated through common pathways, each of the promoters has specific regulation as a consequence of a signaling network, which enables fine-tuning of the system and urokinase activity according to the physiological needs.

## 1. Introduction

**Figure 1:**
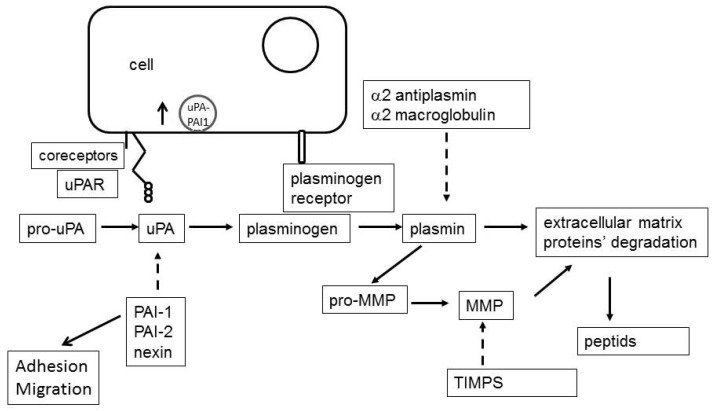
Urokinase plasminogen activation system functions. pro-uPA and PAI1 are secreted from the cell, where uPA is activated and cleaves plasminogen into plasmin, a strong protease which degrades extracellular matrix proteins and activates metalloproteinases. PAI1, besides uPA inhibition, regulates processes of cell adhesion and migration. uPA and PAI1, bound to uPAR, can be endocytosed. uPA: urokinase plasminogen activator; uPAR: urokinase plasminogen activator receptor; PAI1: plasminogen activator inhibitor; PAI2: plasminogen activator inhibitor 2, pro-uPA: urokinase plasminogen activator proenzyme; pro-MMP: Matrix Metalloproteinase proenzyme; MMP: Matrix Metalloproteinase; TIMPS: Matrix Metalloproteinase inhibitor. −−→ inhibition.

The main role of the urokinase plasminogen activation system is the proteolytic activation of plasminogen and production of plasmin, a highly potent protease that can direct different physiological processes in the organism. As plasminogen is present in the serum, the activity of urokinase plasminogen activator or urokinase is the main regulator of plasmin production. Plasmin is involved in the degradation of extracellular matrix (ECM) components during tissue reorganization, such as mammary gland involution after lactation, wound healing, liver regeneration, blood clot resolution, embryo implantation in the uterus endometrium, as well as in tumor metastasis. Plasmin can be involved directly or activate metalloproteases to take part in ECM degradation. Besides these processes, the urokinase plasminogen activation system can also influence cell adhesion, migration, proliferation and cell signaling pathways. The plasminogen activation system comprises urokinase (uPA), an extracellular serine protease, its inhibitor PAI1 and membrane receptor uPAR. Plasminogen can also be activated by the related tissue plasminogen activator. In addition to PAI1, urokinase can be inhibited by PAI2 and nexin [1,2] (Figure 1).

Urokinase is produced and secreted from the cell in the form of prourokinase, which is specifically cleaved by plasmin and some other enzymes extracellularly, to produce two amino acid chains bound by disulfide bonds. Extracellularly, urokinase can bind to its receptor to localize its activity. It can also be cleaved further to produce catalytically active low molecular weight uPA, which does not bind to the receptor. uPAR is anchored to the membrane by a GPI anchor and can bind uPA, its inhibitor and vitronectin. PAI1 binding to uPA on the receptor triggers internalization of the complex and uPA degradation. uPAR was shown to have some additional functions, such as regulation of cell adhesion by interactions and competition between PAI1, uPAR and vitronectin. It cooperates with coreceptors such as Lrp1 and integrins and can be involved in the regulation of proliferation through EGFR signaling. uPAR can also be shed from the membrane and appear as suPAR, the soluble form, in the serum. PAI1, by binding to uPA, inhibits its enzymatic reaction. Therefore, net uPA activity is dependent on uPA and PAI1 secretion and their balance. It is considered that both of them have constitutive secretion, but PAI1 can also be found in storage vesicles, as well as in extracellular vesicles [1,3,4] (Figure 1).

All members of the uPA system are tightly regulated at the level of transcription, but also posttranscriptionally, by miRNA and 3’UTR binding proteins’ regulation [5,6,7,8]. Urokinase, receptor and inhibitor are produced only in some cell types, and urokinase activity can be induced in specific conditions, mostly by specific growth factors. One example is the regulation of uPA and PAI1 during wound healing in keratinocytes, where there is a sequence of processes, including cell migration and proliferation, leading to tissue reparation [9,10]. PAI1’s regulation was found to be coordinated with genes involved in epithelial–mesenchymal transition [11]. As urokinase can be produced by some tumors and can be involved in invasion and metastasis processes, its regulation was the topic of investigation already in the 70ties and 80ties of the 20th century. Some of the first experiments showed an increase in urokinase activity and its expression by phorbol ester, a known protein kinase C (PKC) activator, in different cell lines. Further, several pathways leading to transcriptional activation were described, mainly involving transcription factor AP1 binding to the *urokinase* promoter/enhancer [1]. In parallel, *PAI1* transcription can be regulated by the same common pathways. However, the final outcomes are dependent on the cell type specificities and conditions of the cell microenvironment.

## 2. AP1 Transcription Factor and Its Regulation

### 2.1. AP1 Transcription Factor Structure

**Figure 2:**
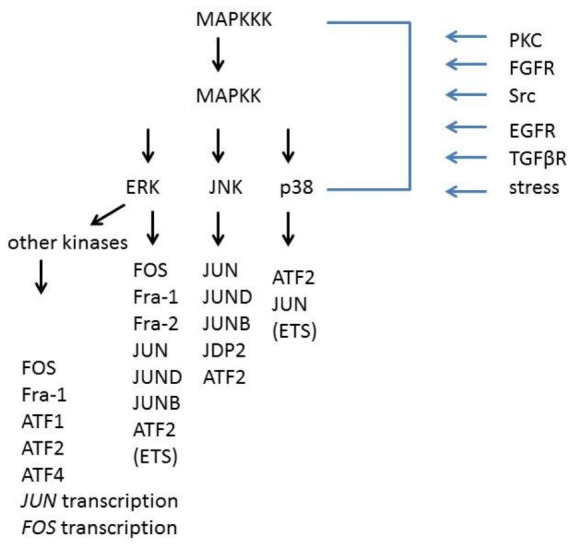
Regulation of AP1 members: upstream signals, such as activation of growth factor receptors, protein kinase C or Tyrosine kinase Src, activate MAPKKK, which, through MAPKK, activates ERK, JNK and p38, as downstream MAPK. Each of these kinases directly or indirectly phosphorylates a different set of AP1 transcription factors, shown below. MAPKKK: MAP kinase kinase kinase; MAPKK: MAP kinase kinase; PKC: protein kinase C; FGFR: Fibroblast growth factor receptor; EGFR: Epidermal growth factor receptor; TGFβR: Transforming growth factor β receptor.

AP1 is a common transcription factor regulating gene expression in response to different stimuli, from growth factors and cytokines to stress agents. It is present in the gene promoter regions, but also in the distal enhancers and is involved in long-range interactions [12]. AP1 is a dimer of two proteins belonging to basic leucine zippers (bZIP), which dimerize through a leucine zipper motif. The first AP1 proteins identified were FOS and JUN, found as viral oncoproteins inducing sarcomas in birds and mice [13]. Later on, it was found that AP1 is a dimer formed from members of the FOS and JUN gene families, but which can also include some members of the ATF and MAF families. JUN family members include JUN, JUNB and JUND. JUN can form homodimers or heterodimers with JUNB, JUND or FOS family members, which comprise FOS, FOSB, FOSL1 (Fra-1), FOSL2 (Fra-2). Members of ATF family include ATF2, ATF3, ATF4, ATF5, ATF6B, ATF7, BATF, BATF2, BATF3 and JDP2. ATF factors can form homodimers and heterodimers. JDP2 is a Jun dimerization protein that can act as a potent repressor of AP1-dependent transcription, recruiting histone deacetylase HDAC3 [12,14]. MAF family includes cMAF, MAFA, MAFB, MAFF, MAFG, MAFK and Nrl [12,15]. Some MAF family members can form dimers with both JUN and FOS (Maf and Nrl) or only with FOS (others) [16]. Different combinations of dimers can have different affinities toward promoter response elements. Different AP1 members can have different, cell-specific expression, and their binding to DNA and activation can be separately regulated through specific phosphorylations. Their DNA-binding domain binds to a consensus binding site TGAg/cTCA. The sequence typical for JUN homodimers and JUN-FOS family heterodimers’ binding is also called TRE, after TPA or 12-O-tetradecanoylphorbol-13-acetate or phorbol ester response element, according to the experiments in which it was discovered. When bound to ATF2, 3 or 4, JUN dimers can bind to the TCACGTCA sequence, a cAMP response element CRE [15]. MAF dimers bind to MARE I or MARE II binding sequences, and were not thoroughly investigated in the context of AP1 regulation [12,15] (Figure 2).

AP1 is involved in the regulation of numerous processes in the cell, from proliferation, invasion and migration, stress response, apoptosis, metabolism, senescence, to tumorigenesis. Genome-wide studies revealed that it can be found in enhancers and superenhancers and is regulated through developmental processes. AP1 was found to mediate regulation of dynamics in chromatin architecture during epidermal cell differentiation in the skin; it was also found to be involved in vasculogenesis during embryonal development, endothelium regulation, hematopoiesis and neural development [17,18,19,20,21]. Their role in higher chromatin organization was also shown, as they were involved in the expression of some cancer genes [22,23].

AP1 complex containing JUN is involved in the expression of genes positively regulating proliferation. JUN can increase cell proliferation depending on MAP kinase activation. It regulates the expression of cyclin D1, but also inhibits negative cell cycle regulators, such as p53 and INK4A [24,25]. FOS is known as an immediate early gene, expressed soon after growth factor stimulation of the quiescent cells, dependent on the ERK activity [26]. JUNB and JUND can negatively regulate proliferation, but JUND effects are cell-type specific [16]. AP1 can also have dual influence on apoptosis, in dependence on dimer composition and cell type [16].

AP1 complex has been considered the master control factor of cell invasion [27]. Genes regulated by it include those directly regulating invasion, like cytoskeletal elements, extracellular matrix proteins and adhesion and indirectly, growth factors. Many of these genes were found to have complex promoter sequences with different combinations of AP1 and ETS, such as in promoters of *metalloproteinases* and *urokinase* [28]. AP1 is also involved in the process of epithelial–mesenchymal transition [29,30].

AP1 members were found to be increased in different types of tumors. Overexpression of JUN and FOS participates in tumorigenesis because of their strong transactivation activities [30]. The oncogene activity of AP1 depended on the composition of the dimers, but also on the intracellular milieu and signaling network in the tumor cells [16]. JUN, FOS and FOSB have strong transformation ability, Fra-1 and 2 have weak, while JUNB and JUND, although considered nononcogenic, can be increased in different types of tumors and have both tumor suppressor and oncogene roles depending on the cell type [16,31,32,33]. As oncogenes, AP1 members were found to have increased expression in different types of tumors, i.e., JUN in squamous cell carcinoma and Fra-1 in colon and breast carcinoma [27]. However, overexpression of v-FOS and dominant negative JUN expression arrays showed some differences, indicating finely regulated processes. In many tumors, AP1 upregulation was a consequence of constitutively active upstream signaling. In addition, AP1 members can cooperate and be coregulated through other signaling pathways and downstream transcription factors, such as ETS, NFκB and TCF/LEF downstream of Wnt signaling [27].

AP1 members were found to be involved in different developmental processes, such as angiogenesis, modeling of vascular endothelial cells’ chromatin landscape and blood cell lineage development [16,19,34,35,36]. AP1 was found to be important in maintaining somatic cell fate and was able to prevent fibroblast reprogramming into induced pluripotent stem cells [37]. Furthermore, it was found to label the enhancer landscape and participate in the transcriptional program of senescence [38]. Part of that program is also the urokinase system, as PAI1 is among the secreted proteins characteristic of senescent phenotype.

### 2.2. Regulation of AP1 Transcription Factor

AP1 regulation can be exerted through several mechanisms. One of them is dimer composition, which depends on the availability and expression of different *FOS*, *JUN* and *ATF* family genes. Another mechanism is their activation and stability, regulated through different signaling pathways and affinity for binding to promoter response elements. Each AP1 protein member has specific dimerization abilities. AP1 can have at least 18 different combinations from members of different or the same transcription factor family [13,39]. Dimers can have different affinities toward specific binding sites. Certain combinations can be important for some specific processes, such as proliferation or anchorage-independent growth [40]. Each component of AP1 has a dimerization domain responsible for leucine zipper formation and a transactivation domain. The latter can be phosphorylated on different positions, and these modifications regulate its transcriptional activity. In addition, cell and signaling context can determine the role of each monomer TF, so the same TF can have opposite functions in different cells. As an example, JUNB can act as a tumor suppressor and an oncogene in B-cell leukemia and Hodgkin lymphoma, respectively [41,42]. FOS and JUN dimers can form contacts with flanking regions, and AP1 binding can be regulated by chromatin context, presence of histones and DNA bending. All these features can allow AP1 to bind to sequences with small differences from the canonical TRE or CRE sites [12,43]. In addition, it was found that AP1 dimers can recognize motifs modified by cytosine methylation [44]. Methylated domains adjacent to the AP1 binding site can also influence its regulation [45]. Even cellular redox homeostasis can possibly influence molecule conformation and dimer formation [46]. It is considered that AP1 can act as a pioneer transcription factor and take part in chromatin remodeling and recruit the BAF chromatin remodeling complex to open chromatin [47,48]. On the higher level of organization, AP1 sites were found in enhancers in chromatin loops, contributing to three-dimensional chromatin regulation [49].

AP1 can form protein–protein interactions and cooperate with other adjacent TF [15]. AP1 was found to cooperate with TEAD family members, recruiting YAP/TAZ involved in the regulation of organ size and tumorigenesis [50]; JUN was found to form complexes with ETS in different combinations [51,52,53,54]. *uPA* promoter is one of several genes with specific composite AP1/ETS (AP1/PEA3) regulatory elements in the promoter [55]. Oncogenic ETS family and ERK signaling can influence binding of different dimers to the neighboring AP1 site and differently regulate expression of target genes in different cell lines [28]. AP1 can also cooperate with NFAT, SMAD and bHLH families, as well as with HNF4A (hepatocyte nuclear factor) binding to sites adjacent to TRE/CRE [15,56]. JUN has been shown to have protein–protein interactions with family members of NF-κB, Sp1, GATA, STAT, glucocorticoid receptor, pRB, CBP/p300, SMRT and other transcription factors [15,57,58,59,60]. AP1 signaling can be inhibited by transrepression through glucocorticoid and retinoic acid receptor signaling, but in some cell types, these can synergize with AP1 [16,61]. In addition, AP1 members were found to cooperate in huge enhanceosome complexes, such as that involved in interferon β regulation [62].

Besides dimerization, AP1 elements are regulated at the level of their own transcription and phosphorylation; the latter regulates their binding to DNA. Some monomers are unstable and depend on the upstream signaling. In this way, FOS can “recognize” the duration of the upstream signaling and only sustained ERK activation can induce transcriptional activities [63,64,65].

One of the main signaling pathways regulating AP1 is the MAP kinases, activated by mitogens, growth factors, cytokines, stress, activation of G protein receptors and calcium [66]. ERK, mainly regulating cell proliferation, differentiation and motility, directly phosphorylates ETS (as a common AP1 interacting factor), as well as FOS, Fra1, Fra2 and JUN, among others, and inhibits FOXO3. Indirectly, it can activate CREB and ATF1, as well as FOS and ATF4 [67,68]. The JNK pathway is regulated by growth factors, but also stress signals. In the nucleus, it can activate JUN, JUND, ATF2, ELK1 and other transcription factors [67]. The third MAPK linked with AP1 regulation is p38. It is often activated in conditions of cell stress and can regulate apoptosis. Also, it is linked with the differentiation of specific cell types. Direct downstream transcription factors’ targets are ETS1 and ATF2, in addition to CREB, activated through downstream kinases [67]. In addition, different AP1 members can have multiple regulatory pathways [67]. JNK and ERK both phosphorylate JUN on different serins. GSK3 and CKII can prevent JUN binding to DNA, but ERK can abrogate this inhibition [63,69]. JUN can also be regulated through some other kinases, such as p21-activated kinase 2 (PAK2), Vaccinia related kinase (VRK) and C-terminal Src kinase (CSK), as well as with PKC [70,71,72,73,74,75,76]. FOS, besides ERK, can also be regulated through kinases RSK2, PKA, Frk and PKC/Cdc2. ATF2 is mainly regulated by p38 and JNK signaling [66,67] (Figure 2).

## 3. Urokinase Plasminogen Activator Promoter

### 3.1. Regulation of the Urokinase Plasminogen Activator Promoter

**Figure 3:**
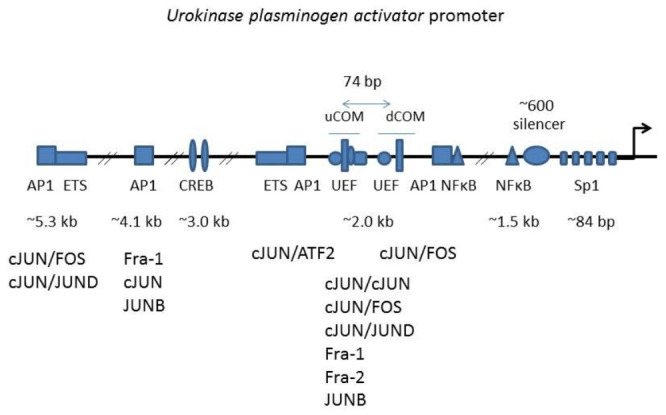
*Urokinase plasminogen activator* promoter. DNA binding sites are shown below the promoter line; the position of the sites is shown as the distance from the transcription start point, and AP1 monomers and dimers bind to AP1 sites shown below. uCOM: upstream cooperation mediator; dCOM: downstream cooperation mediator; UEF: urokinase enhancer factor; AP1, ETS, CREB, NFκB, Sp1: binding sites for corresponding transcription factors. The arrow signs the transcription start point.

**Figure 4:**
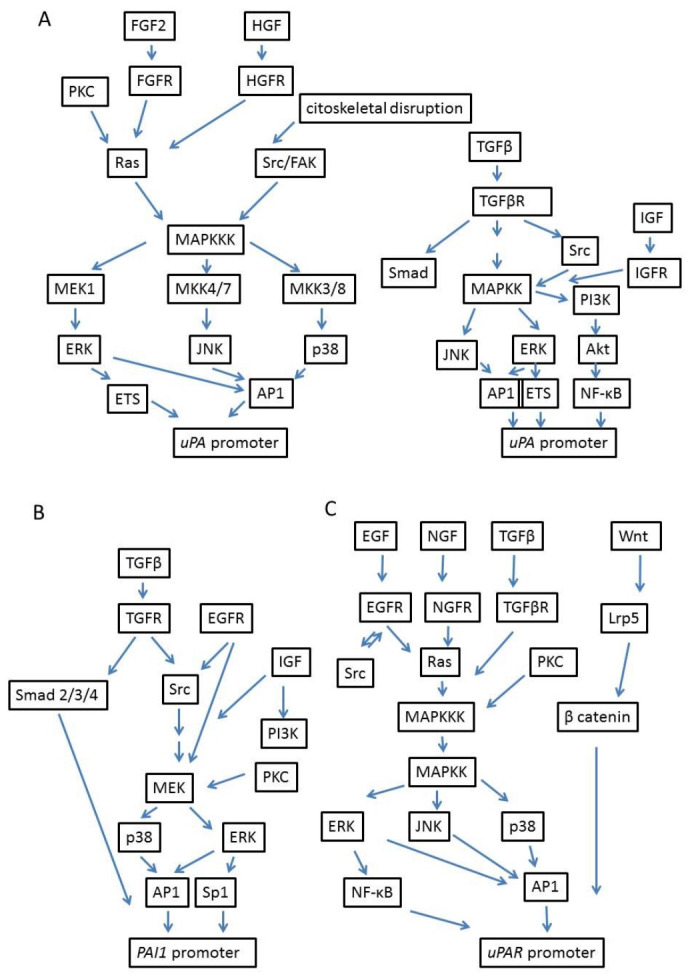
(**A**): Signaling pathways involved in the regulation of *the uPA* promoter. (**B**): Signaling pathways involved in the regulation of *the PAI1* promoter. (**C**): Signaling pathways involved in the regulation of *the uPAR* promoter. All promoters with AP1 and cooperating sites are activated through a cascade of three MAP kinases, MAPKKK, MAPKK and MAPK, JNK, ERK and p38. Upstream pathways activating the *uPA* promoter AP1 are PKC, FGF, HGF and TGFβ pathways. Pathways activating the *PAI1* promoter AP1 are TGFβ, Src and PKC. The *uPAR* promoter AP1 can be activated through activation of EGFR, TGFβ and NGFR. MAPKKK: MAP kinase kinase kinase; MAPKK: MAP kinase kinase; MEK1, MKK4/7, MKK 3/8: MAP kinase kinase; PKC: protein kinase C; FGF: Fibroblast Growth Factor; FGFR: Fibroblast Growth Factor Receptor; EGF: Epidermal Growth Factor; EGFR: Epidermal Growth Factor Receptor; TGFβ: Transformation Growth Factor β; TGFβR: Transformation Growth Factor β Receptor; NGF: Nerve Growth Factor; NGFR: Nerve Growth Factor Receptor; HGF: Hepatocyte Growth Factor; HGFR: Hepatocyte Growth Factor Receptor; IGF: Insulin-like Growth Factor; IGFR: Insulin-like Growth Factor Receptor; uPA promoter: urokinase promoter with binding sites for transcription factors AP1, ETS and NFκB; *PAI1* promoter: promoter of plasminogen activator inhibitor 1 with binding sites for AP1, Sp1 and Smad 2/3/4; *uPAR* promoter: urokinase plasminogen activator receptor promoter with binding sites for AP1, NFκB and β-catenin.

Urokinase is encoded by the gene *PLAU*, located on the 10q22 chromosomal region. *PLAU* promoter can be downregulated by DNA methylation and is not active in many cell types [77]. Also, different cell types produce it in specific conditions, according to their function. So, it can be presumed that it is regulated by some tissue-specific transcription factors or a cell-specific signaling network [78]. Numerous experiments were done to analyze the *uPA* promoter and enhancer region, usually comprising the region of 5–6 kb upstream from the transcription start site [78]. The most analyzed and dominant motifs in *uPA* promoter/enhancer are those of AP1/PEA3 (ETS) in combination with neighboring AP1, present on around 1.9 bp and 5.3 kb upstream of the transcription start [79,80]. Between AP1/PEA3 and AP1 motif, a COM region was detected, possibly binding repressors and HOX-coregulatory factors. The proximal promoter also contains five Sp1 binding motifs at the region of 86 bp upstream of the TATA box, regulated by MAPK/ERK and JNK signaling [81]. Sp1 was also shown to recruit GATA6 [82]. In addition, promoters can be regulated by pathways activating NFκB and those of protein kinase A through cAMP and CREB. The latter were analyzed in porcine kidney cells, where they showed specific hormone-dependent regulation [83]. *uPA* promoter was shown to be activated by MAP kinases ERK, JNK and p38, PI3K/Akt pathway, activation of NFκB and NOTCH signaling. Wnt pathways can lead to ZEB1 expression in colorectal carcinoma cells and regulate *uPA*, possibly through ZEB1 and E box or E box-like sequences [84,85]. All these pathways were activated in different cell types by different stress-inducing agents, by hypoxia, growth factors, hormones, etc. [86,87]. Activation of p53 was also found to regulate transcription of *uPA* [88]. In addition, in the *uPA* promoter, negative cis-acting sequences were found on several sites [80,89] (Figure 3 and Figure 4A).

### 3.2. AP1 Regulation of the PLAU Promoter

Analysis of the *uPA* promoter region showed the existence of several AP1 binding regions in specific organization, in mouse, human and porcine genes [90,91]. The binding sites consist of PEA3 (ETS)/AP1 and an additional AP1 site with a region of around 70 bp between them, named COM. PEA3/AP1 sites were positioned at around 2 kb and in the region of around 5.3 kb upstream of the transcription start (position depends on the species) [79]. Signaling pathways activating these AP1 sites mostly involved MAP kinases, JNK or ERK or both of them [90,92]. Numerous different growth factors and activation of membrane receptors, as well as cell treatment with stress agents, were shown to regulate urokinase expression through downstream ERK and/or JNK pathways and different combinations of AP1 dimers, in a cell-specific way and with different kinetics.

Early experiments showed *urokinase* transcriptional induction in different cells by signaling induced by phorbol esters, disruption of cytoskeletal structures, such as actin and microtubules, inhibitors of tyrosine and serine phosphatases and Fibroblast Growth Factor 2 (FGF2). Phorbol ester [TPA], as a protein kinase C activator, and FGF2, by binding to its receptor with Tyr kinase activity, activated cooperatively both PEA3/AP1 sites (ETS/AP1), which contained binding sequences for AP1 and a member of the ETS family, a transcription factor activated by the ERK pathway. The pathways activated were Ras/Raf/MEK/ERK or MEKK1/MKK1(MEK1)/ERK and MEKK1/MKK4/JNK, depending on the cell type (Figure 3 and Figure 4A) [90,92,93]. Proximal PEA3/AP1–AP1 sites were found to bind JUN homodimers, JUN-JUND, JUN-FOS, JUN-ATF2, as well as Fra1, Fra2 and JUNB, in addition to ETS2 (binding to PEA3/ETS site) in conditions of constitutive uPA expression in mouse fibroblasts and different types of cancer cell lines [79,94,95]. The same factors were found bound after induction with TPA and FGF2, although an increase in ATF2 binding and a decrease in FOS binding were detected [79,94,96,97]. The Distal AP1/ETS-AP1 site was also activated by MAP kinases and bound JUN-FOS and JUN-JUND, in cooperation with the proximal site [79]. Cytoskeleton reorganization of both actin and microtubules induced *uPA* through Ras/ERK signaling pathway, as a consequence of Focal adhesion kinase (FAK) and Src activation, and further JUN phosphorylation [90,98,99]. However, Silberman [100] showed that activation of both ERK and JNK pathways led to a urokinase-expressing phenotype of Ras-transformed mouse fibroblasts, while ERK activation and suppression of JUN and JNK led to downregulation of urokinase expression, but upregulation of cathepsin.

The Ras/Raf/ERK pathway was also induced by Hepatocyte growth factor/scatter factor (HGF), activating Met tyrosine kinase receptor. Basal and induced promoter activation was dependent on FOS and JUND in one cell type, and JUNB in another [101,102]. Similar pathways were also activated by colony-stimulating factor 1 (CSF1) in macrophages and IL-1, alone or in synergy with TPA [103,104]. Following IL-1 activation of JNK, ATF2, JUN, and JUND were found on the uPA promoter [105]. Inhibition of Tyr and Ser phosphatases activated FOS/JUN or only JUN dimers [91]. On the other side, Seddighzadeh [106] found okadaic acid to stimulate ERK activity, increase JUNB expression and decrease expression of *uPA* mRNA in breast cancer cells.

JNK and p38, as stress kinases, were found to activate AP1 sites after inducing different types of stress and DNA damage in the cells. uPA expression was induced in several DNA repair-deficient human cells by DNA-damaging agents, such as UV in xeroderma pigmentosum cells, or by alkylating agents in O6-methyl guanidine methyl transferase-deficient cells [107,108,109]. UV irradiation induced *uPA* through JNK and JUN activation and AP1 binding, but also p38 and FOS binding in another setting [110]. Doxorubicin treatment of lymphoma cells acted through MEK1/2 and p38 MAPK pathways [111].

Many cancer cell lines have constitutive uPA expression. In squamous carcinoma cell lines, ERK activation of the AP1/PEA3 site involved binding of FOS and JUND [112]. EGFR phosphorylation-activated JNK induced *uPA* through an autocrine loop in the prostatic cell line [113]. Chen [114] found the MK2 signaling pathway activating p38 and ERK to be responsible for high AP1 activity in breast cancer cells. p38 also phosphorylated JAB1, JUN activation domain-binding protein, which is a transcriptional coactivator of JUN and stabilizer of AP1 complex, and ERK was responsible for the high expression of JUN. In liver cells, *urokinase* expression was dependent on Fra1 binding at ~2 kb AP1 site, and in aggressive breast cancer cells at ~2 kb (−1.9 kb) and −4.1 kb position. While FOS and FOSB were not expressed in cells analyzed, JUN, JUNB, JUND and Fra2, besides Fra1, were found on both AP1 sites [95,115]. AP1 combinations on the *uPA* promoter and their roles, in basal or induced conditions, depended on the cell type. While in some cells overexpression of JUNB did not have an influence on uPA expression, lack of JUNB caused embryonic lethality in mice, in the period in which wild-type extraembryonic tissues showed their high expression. It is supposed that the JUNB deficit led to deregulation of uPA expression, among other molecules, and caused defective neovascularization of the decidua [116].

AP1 sites in the *uPA* promoter were found to cooperate with each other and with other binding sites and transcription factors. Besides cooperation between ETS and AP1 in composite binding sites, cooperation between proximal and distal AP1 sites was also found [108,114]. A regulatory element, cooperation mediator (COM), is required for cooperation between PEA3/AP1 and the neighboring AP1 site in the enhancer. The COM region (74 bp) consists of several overlapping binding sites for UEF 1–4 factors, some of which are highly conserved. The region has a bipartite structure, consisting of upstream and downstream regions [55,117,118]. Four protein complexes were found bound to the COM sequence (UEF4, UEF3 and UEF1 upper and lower complexes). On the UEF3 site, a complex of three polypeptides was found, containing different combinations of Prep-Pbx proteins (Prep1+/−Pbx1a or Pbx2; or Prep1+/−Pbk1b). Prep1 and Pbx are important HOX partners during developmental processes, but in the *uPA* promoter, they were found to act independently [118]. The UEF4 binding site overlapped with UEF3 and bound ubiquitous transcription factor Oct1, which can disturb the binding of the Prep-Pbx complex on the UEF3 site. Increased binding of Oct1 can decrease basal promoter activity. The sequence can also bind Oct2, specifically expressed in B cells and neuronal cells [118]. It is supposed that UEF1, UEF3 and UEF4 complexes are functionally redundant. At the same time, COM binding sequences are overlapping and different sets of proteins are supposed to bind to the region in basal and induced conditions. The COM sequence was involved in the uPA induction by TPA and participated in cooperation between PEA3/AP1 and glucocorticoid response element [55]. Its disruption can lead to loss of promoter induction by TPA, but its partial deletion increased the basal transcription [117].

The complex regulation of the AP1/PEA3 site was explored in experiments with prostate cell lines done by Selvaraj et al. [28]. They showed that different JUN family members can regulate cell migration through a subset of extracellular proteases, which contained complex AP1-ETS sites in the promoters. In the basal conditions, JUN was a stronger activator of its promoters than JUND, but in the conditions of ERK signaling, phosphorylation of JUND and ETS made JUND a stronger activator than JUN. ETS phosphorylation increased its affinity for the CBP/p300 coactivator and upregulated transcription. It is supposed that JUND is involved in this way in the regulation of the cell migration-regulating genes, and not those involved in proliferation, which can be downregulated. In addition, oncogenic ETS factors cooperated with JUN, even in the absence of Ras/ERK signaling and upregulated transcription. In some cell lines, oncogenic ETS pathways were regulated through the PI3K/Akt pathway. Therefore, neighboring ETS proteins can alter the function and affinity of AP1 members and regulate transcription in dependence on the signaling pathways.

Besides MAP kinases, other signaling pathways were also shown to participate in the regulation of the AP1 site. Dunn [119] showed that IGF1 induced *uPA* in breast cancer cell lines, signaling through both PI3K and MEK/ERK pathways and involving AP1 and ETS transcription factors. Stromal cell-derived factor binding to chemokine receptor CXCR4 induced *uPA* through p38 and PI3K pathways and downstream Sp1 and AP1 binding in colon cancer cells [120]. Also, in lung cancer cells, it was shown that EGF signaling was transduced through Akt activation, upregulating urokinase expression [121]. β-catenin was shown to regulate the human *uPA* promoter directly via TCF-4 and two TBE (TCF4 binding element at around −737 and −562 bp) in synergy with AP1 and ETS1 [85]. However, in another cell type, overexpression or accumulation of β-catenin decreased uPA and uPAR expression, but was regulated through the NF-κB pathway [122]. In multiple myeloma cells, cell migration was influenced by CD40 signaling, activating both the MAPK/ERK pathway and PI3K-Akt-NF-κB; but, the latter regulated urokinase expression [123]. In addition, one NF-κB site was found close to the proximal AP1 site and cooperated with it in a TPA-induced *uPA* transcription [124].

Interactions between AP1 and cAMP signaling also exist. JUN can form dimers with ATF members that belong to the CREB family, and some of them prefer CRE sequences to bind [96]. There were many experiments on pig kidney cells showing uPA regulation through protein kinase A and cAMP. Kidney cells have specific regulation of the *uPA* promoter by LFB3/HNF1B, which cooperates with cAMP response element CREB in the upstream (~3.4 kb) binding site. However, it was shown that HNF1B was involved (negatively) in the crosstalk between cAMP signaling and AP1 regulation, as CREB/ATF could dimerize with AP1 members [125]. Synergistic transcriptional activation of the *uPA* promoter by AP1 and other TF was shown in cAMP and retinoic acid-mediated induction [126]. In myoblasts, PEA3/AP1-AP1 element bound JUN, JUND and ATF2, in cooperation with CRE/CArG element binding SRF [127].

Possibly one of the most intriguing regulations of the *uPA* gene was shown by TGFβ treatment. All uPA system genes’ transcription can be TGFβ-responsive. TGFβ binding to its receptor can activate, besides canonical Smad transcription factors, also Src, ERK1/2 and Akt pathways, as well as JNK [128]. Two signaling pathways are supposed to be involved in the uPA regulation, Src-MAPK-PI3K-NF-κB and Src-MAPK-AP1 [129], as the *uPA* promoter contains sequences binding NF-κB as p50/p65 and cRel/p65 dimers [130]. Therefore, the same upstream pathways can activate either NF-κB or AP1 site, or both, in a cell-specific way [131]. Some uPA inhibitors, such as epigallocatechin gallate, genistein and others, were found to act through inhibition of both AP1 and NF-κB activation [132,133]. Signaling induced by integrin activation can also lead to urokinase regulation, through different pathways, in a cell type-specific way, either involving Src, ERK/AP1 and JUN phosphorylation, cooperation with Akt pathways or using NF-κB (Rel) pathways [134,135,136] (Table 1).

Finally, in some settings, it seems that AP1 regulation, leading to urokinase modulation in parallel with other extracellular proteases, is a part of the global chromatin regulation and programming. Starvation and depletion of growth factors and serum in fibroblast cell culture lead to modification of JUN histone marks (H3K27ac). In the pro-proliferation state, both JUN and FOS members changed histone marks [139].

## 4. Plasminogen Activator Inhibitor 1 Promoter

### 4.1. Regulation of the Plasminogen Activator Inhibitor 1 Promoter

Urokinase activity depends on both urokinase and PAI1 expression and secretion, as PAI1 decreases urokinase activity by its inhibition. Besides this role, PAI1 can regulate cell adhesion and migration through the interactions with uPAR and vitronectin and competition with integrin αVβ3 [140].

PAI1 is regulated through numerous growth factors, hormones and stress signals; many of them also regulate uPA [1]. The most explored pathways of PAI1 regulation are those of TGFβ and hypoxia, as well as those involved in its regulation by insulin and senescence [141]. In these conditions, PAI1 regulation seems to be a part of the complex signaling network.

PAI1 is encoded by the gene *SERPINE1*, located on chromosome 7 (7q21.3-q22). Its promoter is known for the presence of a polymorphism known as 4G/5G, where the 5G variant is transcriptionally less active [142]. The most prominent pathway involved in *PAI1* regulation is TGFβ signaling. Three TGFβ-dependent responsive elements interacting with Smad transcription factors were found in the region from around −286 to −800 bp from the start position [143]. The canonical TGFβ pathway starts from TGFβ binding to its receptor and activation of Smad proteins. Trimers made of common Smad 4 and different combinations of activated Smad 2 and 3 bind to specific promoter regions. However, it was found that TGFβ signaling is extremely complex and dependent on the context, from activity of different pathway inhibitors, dependence on the receptor localization, to promoter binding [144]. It was found that the *PAI1* promoter can be regulated by TGFβ, but not only through direct binding of Smads to their response elements, but also by influencing other regions, i.e., proximal Sp1 binding regions can bind Smad in combination with Sp1 [1,145]. TGFβ was found to regulate binding to CTF/NF1 and E-abox/USF (ubiquitous factor) sequences, as well as synergistic binding of TFE3 and Smad3 in cooperation with CBP/p300 [146,147]. As activated TGFβ receptor can, besides Smad pathways, also induce MAP kinase pathways, in some cells, the pathways chosen regulate whether PAI1 will be upregulated or downregulated [148]. In addition, some transcription factors are negative regulators of TGFβ-dependent PAI1 induction, such as KLF2 and glucocorticoid signaling [141].

**Figure 5:**
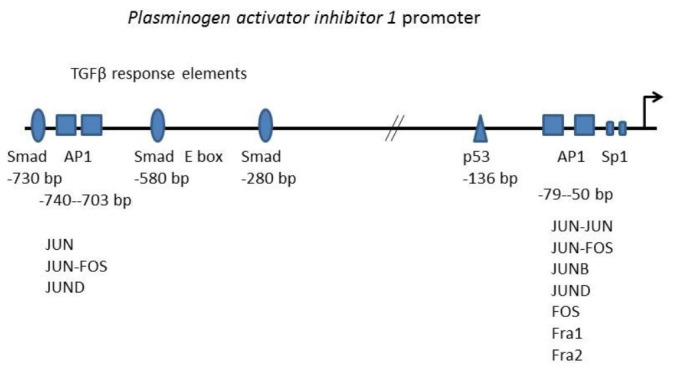
*Plasminogen activator inhibitor 1* promoter. DNA binding sites for transcription factors AP1, Smad, p53 and Sp1 are shown below the promoter line; The position of the sites is shown as the distance from the transcription start point. AP1 monomers and dimers binding to AP1 sites are shown at the bottom.

PAI1 regulation seems to be a part of global changes in transcription following changes in physiological conditions, such as serum stimulation of G_0_ keratinocytes. This process was found to be dependent on the different regulation of E-box motifs in the distal region, in quiescent and serum-activated cells. This region was suggested to act as a platform for recruitment of positive and negative regulators (such as different upstream stimulatory factors (USF, TFE3, SMAD, etc.). A number of interactions regulate the outcome, such as those between USF factors and YY1, Smad and YY1, exchange USF1 to USF2 or the influence of EBPβ, depending on the upstream signaling [149,150,151,152,153] (Figure 4B and Figure 5).

Another PAI1 upregulator is hypoxic condition. In the conditions of low oxygen, the level of HIF1 transcription factor, a dimer of HIF1α and β, is increased due to HIF1α stabilization. It can bind to HIF1 recognition sequences (HRE) in the proximal regions of the *PAI1* promoter, but also cooperate with proximal Sp1 and Sp3 binding regions, in a cell-specific way [154,155,156]. Hypoxic conditions were also linked with the regulation of PI3K and ERK, influencing the *PAI1* promoter [154,157]. On the other side, the HRE binding site can be activated by CREB family members binding, after activation by glucagon/cAMP signaling, independently of the oxygen conditions [157]. HIF1 binding to the HRE1 site was also found to compete with USF2a in hepatocytes and Net, a member of ETS transcription factors [158,159,160].

PAI1 is often upregulated in type 2 diabetes or insulin resistance syndrome. Its regulation is specific during the differentiation of adipocytes, whose metabolism is regulated by insulin [161]. Although diabetic adipocytes lose some metabolic responses to insulin, some promoters still respond to insulin signaling, such as that of *PAI1*. In functional cells, insulin, through interactions with its receptor, activates PI3 kinase and MAP kinase ERK, and PAI1 expression is inhibited due to PI3K signaling and partially by E2F activation. Namely, E2F was found to inhibit the *PAI1* promoter and is available during cell proliferation [162]. However, it was found that PI3K signaling was changed in adipocytes and muscle cells with insulin resistance, while ERK pathways kept their function. Therefore, the *PAI1* promoter can be upregulated in such conditions through the MAP kinase pathway. Different regions of the *PAI1* promoter were found to be regulated by insulin: Sp1 activation and binding to proximal binding sites, activation of AP1 and HIF-1 by ERK pathways, in addition to downregulation of E2F inhibition [153,163,164]. PAI1 regulation was also shown to be linked to a signaling network regulated by adipocyte differentiation. In preadipocytes, insulin does not significantly induce PAI1, but after differentiation, the level of free E2F, due to the cells’ nonproliferative state, is low, and insulin can upregulate the PAI1 promoter. Therefore, adipose tissue can be a major PAI1-producing organ [1,162,164]. In addition, PAI1 inducers in adipocytes are TGFβ and TNF alpha, as well as oxidative stress [161].

PAI1 secretion is also increased in the process of cell senescence in fibroblasts, as a part of the senescence-associated secretion phenotype program [165]. In addition, cells under stress can increase PAI1 expression due to p53 activation, and PAI1 can lead to a cell growth arrest in an autocrine way, and promote senescence [166]. AP1 was found to be one of the pioneer senescence enhancers and to help organize the transcriptional network in senescent cells [38]. Besides p53, TGFβ is also involved in the cell arrest regulation in fibroblasts [167]. DNA-damaging agents activating p53 can induce *PAI1* through a p53-responsive element [167,168]. However, Omer [169] found that constitutive stress induced the formation of stress granules in proliferative and presenescent cells. They can inhibit the progression to fully senescent cells and maintain them in a proliferation state. Stress granules recruit *PAI1* mRNA, and thus inhibit its secretion.

There are numerous negative modulators of *PAI1*, such as glucocorticoids and estrogen receptors α and β [170]. β-catenin overexpression was found to oppose NF-κB pathways and to downregulate *PAI1* in some cell types [122]. YAP was shown to be a negative regulator of the Hippo signaling pathway and was bound to the promoter together with TEAD4 in hepatocellular carcinoma [171]. The *PAI1* promoter was also found to be regulated by circadian clock components [172].

*PAI1* has two transcripts, of 3.2 and 2.2 kb, with different 3’UTR regions due to two polyadenylation sites [173]. Several specific proteins bind to the 3’UTR and regulate *PAI1* mRNA stability. TGFβ and insulin pathways can increase the stability of the longer form, and IGF1 pathways of both of them [141].

### 4.2. AP1 Regulation of the PAI1 Promoter

**Figure 6:**
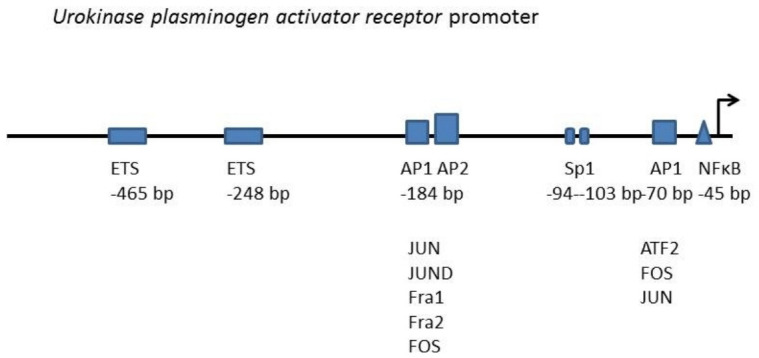
*Urokinase plasminogen activator receptor 1* promoter. DNA binding sites are shown below the promoter line, as well as their position in terms of distance from the transcription start point. AP1 monomers and dimers binding to AP1 sites are shown at the bottom.

The *PAI1* promoter has several AP1 binding sites. In the proximal promoter, these AP1 sites are near Sp1 sites, in the region of 80 to 50 bp upstream of the transcription initiation site. Another region containing AP1 binding sites is distal, at about 800 bp from the initiation site, near regions that bind Smad transcription factors. AP1 sites can be regulated through different MAPK pathways, but also the TGFβ pathway, which is dominant in *PAI1* regulation. Data obtained from different cell types indicate cell-specific cooperation and competition between PI3K, ERK and Smad signaling [174] (Figure 6).

JNK and/or ERK pathways are often involved in the regulation of the proximal two AP1 sites (−58–50 and −79–72 bp), and FOS and JUN were found bound in basal conditions and PKC activation or cytokine treatment, in different cell types [175,176,177,178,179,180,181,182,183]. Also, ATF2/JUN, FOS/JUN and FOS/JUND were found after different types of treatment in different cell types. In breast cancer cells, overexpression of Fra1, Fra2 and FOS increased *PAI1* expression [184].

TGFβ signaling was shown to activate several downstream pathways, including Smad, PI3K, MAPK and β-catenin [185]. Experiments on hepatoma cells showed that MAPK/JNK signaling converged with Smad signaling on the *PAI1* promoter [186]. Downstream pathways and TF binding were shown to be cell-specific. Keeton [186] analyzed an 800 bp *PAI1* promoter, which was fifty-fold activated by TGFβ. Two regions were responsive, proximal (−49 to −87) and distal (−638–740), containing AP1 binding sites; the latter also comprising a Smad-regulated region. Experiments with TRE sequences (TPA responsive gene promoter elements), characteristic for AP1 binding, showed Smad binding, either without AP1 transcription factors or in cooperation with them [187,188]. When Smad2/3 and Smad4 cooperated with AP1 dimers, these processes involved JUN, JUNB, FOS and Fra1 [189,190]. In dependence on the cell type, downstream ERK and JNK activation was also found to be involved [174,191,192,193]. In colorectal cells, for example, the TGFβ-JNK-ATF2 pathway was activated, regulating also epithelial–mesenchymal transition [194]. However, in another cell-specific milieu, TGFβ-induced *PAI1* expression was inhibited by Smad 3/2 overexpression [185].

In hepatocarcinoma cells, TGFβ and EGF synergistically induced PAI1 through MEK1,2 and p38 pathways. EGF pathways were also found to stabilize *PAI1* mRNA. In subsaturating concentrations of growth factors, AP1 and Smad were detected on the promoter [195]. Similar were the results of treatment with TGFβ and IL1β, which stimulated both ERK and Smad3. However, inhibition of TGFβ-activated kinase (TAK1) in the same experiment downregulated PAI1 and downstream processes. TAK1 inhibition decreased transcriptional activity of NF-κB and Smad3 and phosphorylation of JUN, while Smad 1-5-8 signaling was increased [196]. In another setting, serum and TGFβ were found to regulate FOS binding to distal AP1 motifs [149]. However, KLF2, involved in the regulation of endothelial cell quiescence, was shown to downregulate PAI1 expression under basal and TGFβ-induced conditions. KLF2 induced inhibitory Smad7, which influenced TGFβ signaling through Smad2 and through inhibition of JUN phosphorylation [197].

Fibrosis is a pathophysiologic state of ECM accumulation, often linked with PAI1 overexpression. In the disease model conditions, fibroblast and kidney epithelial cells upregulated *PAI1* through AP1 and Sp1. Activation of ERK, JNK and p38, but also Smad2/3 and TGFβ pathways, was found and phosphorylation of JUN, FOS and Sp1. GSK-3 inhibition attenuated activation of MAPK and Smad signaling [198].

PAI1 regulation is linked with insulin signaling. Insulin binding to the insulin receptor relays the signal to the Ras/ERK signaling pathway, but also to PI3K. ERK activation leads to transcription by AP1 and HIF-1 binding, while PI3K signaling can have an inhibitory effect [153,162,164,199]. In adipocytes and skeletal muscles from type 2 diabetes patients, insulin signaling induced activity of ERK and activated FOS, Fra1 and JUN [200,201,202]. AP1 was responsible for PAI1 upregulation under high glucose concentration treatment alone, or in combination with TGFβ [203,204]. In hypoxic conditions, AP1 cooperated with HIF [153]. Stress signaling in the conditions of silica and nickel treatment and oxidative stress mainly involved JNK and ERK, and downstream JUN and FOS [205,206,207]. Glutathione, decreasing ROS, inhibited *PAI1* induced by TGFβ and influenced the binding of AP1, Sp1 and Smad [208].

In *PAI1* proximal part of the promoter, cooperation AP1, containing FOS or JUN, with two Sp1 factors was detected, in the conditions of angiotensin II stimulation and amino acid deprivation [209,210]. However, in another setting, FOS/JUN activation of the promoter also required STAT and NF-κB activation [177].

*PAI1* expression was found to be regulated through the JUN/JNK pathway during the development of different organs, such as during eyelid development [211] (Table 2).

## 5. *uPAR* Promoter

### 5.1. Regulation of the uPAR Promoter

Primarily, uPAR is regulated at the level of the chromatin structure. It has cell-specific expression and can influence cell fate and signaling through interaction with uPA and PAI1. It can, through competitive binding to PAI1, regulate cell adhesion and migration, and through internalization of the uPA-PAI complex, uPA activity. Although inserted into the membrane through a GPI anchor, it can participate in signal transmission through its interactions with integrins and EGFR. It can also be shed from its anchorage and be present in the serum in the form of soluble suPAR. In glioblastoma, it was shown that uPAR can induce a mesenchymal gene expression signature and influence cell survival [215].

The *uPAR* gene (*PLAUR*) is located on 19q13.31 human chromosome, and its promoter region is characterized as around 800 bp long, although it is supposed that the distal region, spanning up to 1500 bp, could also have a regulatory role. It was found that the presence of histone variant H2AZ can negatively regulate promoter [216]. The promoter lacks TATA and CAAT boxes, but contains GC-rich sequences and is responsible for constitutive expression in some cell types [217]. The proximal part of the promoter (up to −184 bp) is rich in Sp1 (2 sites), AP1 (2 sites), AP2 and NF-κB binding sequences. Binding of other TF was also detected, such as KLF4 (3 sites), GATA2, YY1, NFATc1 [218,219,220,221,222,223]. There are also two PEA3/ETS sites in the region from 248 to 465 bp. The proximal promoter site was shown to act as a silencer, mediated by β3 integrin signaling [220]. Constitutive high uPAR expression found in colon cancer cells was regulated through proximal promoter binding of Sp1. Several signaling pathways, such as Src, also regulate the promoter through Sp1 [224]. Posttranscriptional regulation was also found, by binding of different signaling molecules and miRNA to 3’UTR [225,226,227]. Hypoxic conditions in breast cancer cells induced both mRNA stabilization and increased transcriptional activation [228]. (Figure 4C and Figure 6).

### 5.2. AP1 Regulation of the uPAR Promoter

AP1 was found to bind to two regions in the proximal part of the *uPAR* promoter, one in the region around −70 bp, and the other at the position of −184 bp. AP1 binding was found to be responsible for constitutive and induced expression of uPAR in cancer cells. In addition, Wang [229] found AP1 and ETS binding regions and detected JUN and FOS family members in the first intron of the *uPAR* gene (the region 1123/1134), after TPA treatment.

Most of the experiments showed binding of the AP1 complex to the −184 bp sequence. In one colon cell line, under basal conditions, JUN, JUND and Fra1 were bound. TPA treatment caused binding of JUND, JUN and FOS in previously nonexpressing cells, and increased JUN, JUND and Fra1 binding in already expressing colon cancer cells [113,228]. Similarly, JNK and JUN binding were detected in ovarian cancer, but also Rac1 and MEKK1 activation, which participated in the *uPAR* regulation [224,230]. The binding of JUN and FOS family members was also present in the proximal AP1 site in basal conditions of some tumor cell lines and after growth factor treatment [231,232,233]. uPAR was also found to be regulated through Ras and RalA, small GTPases. RalA activated Src, and the pathway caused binding of ATF2-like factor to the AP1 site at −70 bp and JUN to the −184 bp site. Src pathways also activated JNK and caused binding of phosphorylated JUN, JUND and Fra1 to the distal AP1 motif in another setting [220]. Similarly, TGFβ signaling induced activation of JNK, accompanied by Ras and MKK4 and induced JUND phosphorylation. Distal AP1 was also found to bind Fra2 in another cell type [234] (Figure 4C).

Activation of distal AP1 site by different pathways can potentially be synergistically regulated by activation of Sp1 and Ap2 sites at −152/−135, in different cancer types, in basal and after TPA treatment [217,220,235]. TPA and Src signaling were found to involve, besides AP1, also NF-κB [236,237,238,239]. In addition, it was described that the PI3K-Akt pathway can regulate FOS, and consequently *uPAR* [178].

*uPAR* was upregulated by the Wnt pathway in colon cancer [240], as β-catenin induced binding of JUN and Fra1 to the promoter. Direct interaction of β-catenin/LEF complex and JUN/Fra1 was detected. However, Wang [241] found β-catenin/TCF4 to regulate *uPAR* promoter through Sp1 motifs, and β-catenin overexpression inhibited NF-κB signaling in the proximal part of the promoter.

AP1 induction is often linked with stress conditions, leading to *uPAR* upregulation. Aspirin treatment upregulated the distal AP1 promoter site by binding JUN and Fra1 [242], similarly to UV irradiation, cadmium treatment and hypoxic conditions [243,244] (Table 3).

## 6. Discussion

Having an important role in the regulation of extracellular matrix remodeling, cell migration and invasion, the uPA system is strictly regulated, and all its molecules are coordinately modulated in different processes.

Basically, urokinase activity depends on the cell’s ability to express urokinase: promoter chromatin state determines whether cells produce urokinase in basal conditions and whether it can be induced. *Urokinase* has a complex promoter, mainly dependent on the AP1 regulation in combination with ETS, and the promoter is mostly activated by increased binding of AP1 dimers. In general, the *uPA* promoter is regulated through AP1 on several levels. Expression and stability of different AP1 monomers are determined by upstream signaling. Activation of MAP kinases, responding to upstream signals, phosphorylates the available set of AP1 monomers according to their specificity. Different dimers can have different affinities toward the same sequence. Depending on the dimer structure, they can have higher affinity toward TRE or CRE sequences, which can also have uncanonical variants, i.e., CRE binding sites recruit certain types of ATF dimers. In the *uPA* promoter, there are also several potential AP1 sites, not known to bind AP1. However, one of them, at 4.1 kb, was found active in breast cancer, under constitutive expression of Fra1 [95]. Intricate regulation of the complex *uPA* promoter was maybe the best illustrated through the experiments of Selvaraj et al. [28]. In prostate tumor cell lines, in basal conditions, JUN dominated over JUND in binding to the promoter, while in the conditions of ERK signaling, phosphorylated JUND and ETS regulated transcription. In the conditions of oncogenic ETS expression, JUN was the dominant binding factor, and the regulation can possibly depend on the PI3K/Akt pathway. JUND and JUN can therefore have different affinity toward the *urokinase* promoter depending on the regulation and modifications of the neighboring ETS and activity of the signaling pathways. It is interesting that a set of genes with complex AP1/ETS binding sites in promoters was involved in migration. At the same time, JUND binding to other promoters was found to inhibit proliferation [28]. In addition, JUND phosphorylation by ERK and JNK, in contrast to other JUN and FOS members, is influenced by JUND binding to menin, which is “titrating” JUND availability [245]. Promoter regulation by JUNB and FOS also depends on the context and can be involved in both the upregulation and downregulation. Besides promoter regulation through binding of AP1 dimer variants and its affinity regulation through ETS, the COM region between complex AP1/ETS and AP1 adds an additional layer of complexity. This region binds different HOX cofactors and other TF, and, as the binding sites overlap, some transcription factors can compete for binding and additionally upregulate or downregulate AP1 activity on the promoter [117]. However, AP1/ETS site cooperates also with other transcription factors, bound to neighboring sequences, regulated through parallel pathways. Therefore, it seems that urokinase expression depends, besides factors from the cell microenvironment, on a cell-specific signaling network. It should be added that most of the investigations were done on tumor cell lines, which could have deregulated expression or activation of AP1 monomers [246]. Possibly, only small differences in the affinity and relative ambiguity of various AP1 dimers on the binding site enable the fine modulation through multiple inputs from cell signaling pathways.

*PAI1* promoter is dominantly regulated by TGFβ/Smad signaling, but AP1 binding can have a modulatory function on that signaling, possibly dependent on the cell-specific signaling network. It is interesting that *PAI1* can be downregulated as a consequence of proliferative MAPK signaling, through E2F. At the same time, these MAP kinases can influence and upregulate AP1 binding, and, in addition, compete with PI3K pathways [198]. FOS and JUN binding were found in both basal and induced states, and ATF2/JUN and Fra1, Fra2, FOS and JUND were found to increase the expression.

uPAR is the third member of the uPA system. It can influence uPA activity by localizing it in the cell neighborhood and can, by binding PAI1 and triggering uPA-PAI1 endocytosis, regulate cell adhesion and migration. AP1 regulation seems to be involved in the complex interactions with NFκB and β-catenin signaling. AP1 dimers binding to *uPAR* in basal conditions in different tumor cells were found to contain JUN, JUND, FOS and Fra1, and in the induced state JUND, JUN, Fra2, Fra1 and FOS. uPAR was shown to be often upregulated in parallel with uPA [244].

Finally, the net urokinase activity depends on the interaction between uPA and PAI1. Being regulated through basically the same and yet differently modulated transcription factors, involved in numerous signaling loops, enables this system to be finely tuned according to the situation. Several pathways regulate both *uPA* and *PAI1* promoters, such as TGFβ, MAP kinases, Wnt signaling and p53 activation [10,119,174,225]. In addition, regulation can be on both the transcriptional and posttranscriptional level [168,225]. uPA and PAI1 expression can be controlled by the same common transcription factors, such as AP1, Sp1, ETS and others, such as Zeb1, whose role in uPA system coregulation was found in cancer [84]. MAP kinases, but also TGFβ pathways and their downstream signaling, on the other hand, can regulate, through AP1 elements, all three genes of the uPA system. Therefore, AP1 can potentially, in cooperation with other signaling, be a coregulating factor among uPA system molecules, finely tuning the outcome according to the cell’s needs, through dimer composition, affinity of specific promoter binding sites, and intracellular context. However, deciphering the whole mechanism and the signaling network, especially in distinct physiological situations and involving specific cell types, requires much additional investigation.

## 7. Conclusions

It can be supposed that all uPA system elements, uPA, PAI1 and uPAR, are coordinately regulated in “normal” cells by a cell type-specific network, in time and space, dependent on the cues from the cell environment to establish tissue homeostasis. Their expression not only regulates urokinase activity and its consequences in addition to processes of adhesion and migration, but also cell biology through feedback signaling loops from interactions among urokinase, its inhibitor and receptor, as well as through the influence of other extracellular proteases, matrix proteins and membrane coreceptors and receptors, activating intracellular signaling.

AP1 can be considered as one of the key regulators of the uPA system, coordinating the activities and finely tuning the relations between uPA, PAI1 and uPAR. These outcomes can be achieved through modulation of AP1 dimers, their activity and binding with different affinity to promoter binding sites, as well as by cooperation with other transcription factors and pathways. Primarily activated by MAP kinases, they are involved in their intricate positive and negative feedback loops, dependent on the cell specific signaling network and signals received. It is becoming evident that it is not one pathway, but the network of signaling pathways that finely modulate the expression of each of the members and thus the final activity of urokinase, cell migration and adherence ability. Each of the members’ promoters has a different “pattern” of TF binding sites in addition to different partners cooperating with AP1 or its members. The fine mechanisms of the network regulating processes of wound healing and tissue degradation during physiological processes, coordinating cell migration, tissue degradation and proliferation, in time and space, are still to be discovered.

## Figures and Tables

**Table 1:** Regulation of AP1 sites in the urokinase promoter.

Promoter Site	Transcription Factor	Signaling Pathway	Interfering Pathways	Cell Type	References
AP1/ETS-COM-AP1 (~1900 bp)	JUN-JUN JUN-JUND JUN-FOS Fra1 Fra2 JUNB JUN-ATF2	basal conditions		mouse fibroblasts aggressive breast cancer	[79,94,95]
AP1/ETS-COM-AP1 (~1900 bp)	JUN JUND ATF2	basal conditions	CRE element binding SRF	myoblasts	[127]
AP1/ETS-COM-AP1 (~1900 bp)	JUN-JUN JUN-JUND ↓ FOS ↑ ATF2	FGF2 → TPA → PKC → ERK JNK	ETS	mouse transformed fibroblasts keratinocytes	[93,96]
AP1/ETS-COM-AP1 (~1900 bp)	AP1	TPA	COM region ETS glucocorticoid response element	hepatocyte	[55,117]
AP1/ETS-COM-AP1 (~1900 bp)	↑ JUN	FAK-Src-MAPK (→ERK, JNK) (cytoskeletal disruption)	ETS	pig kidney epithelial cells	[90,98]
AP1/ETS-COM-AP1 (~1900 bp)	↑ JUN-FOS ↑ JUN	phosphatase inhibitor Tyr phosphatase inhibitor		mouse keratinocytes pig kidney cells	[91]
AP1/ETS-COM-AP1 (~1900 bp)	JUND	Tyr phosphatase inhibitor (decrease in urokinase expression)		breast cancer cells	[106]
AP1/ETS-COM-AP1 (~1900 bp)	↑ JUN	UV irradiation JNK	cooperation of two AP1 sites	mouse transformed fibroblasts teratocarcinoma	[108]
AP1/ETS-COM-AP1 (~1900 bp)	↑ JUN, FOS, JUND JUNB	HGF-Met receptor	ETS	canine kidney cells mouse fibroblasts	[94,101,102]
AP1/ETS-COM-AP1 (~1900 bp)	↑ AP1	CSF1 TPA	ETS	macrophages	[103,104]
AP1/ETS-COM-AP1 (~1900 bp)	↑ ATF2 JUN, JUND	IL1 → JNK TPA → ERK, JNK	ETS	squamous cell carcinoma	[105,137]
AP1/ETS-COM-AP1 (~1900 bp)	FOS JUND	ERK		squamous cell carcinoma	[112]
AP1/ETS-COM-AP1 (~1900 bp)	↑ AP1	EGFR → JNK		prostatic cell line	[113]
AP1/ETS-COM-AP1 (~1900 bp)	↑ AP1 AP1/JAB1	MK2 → p38, ERK		gastric carcinoma breast carcinoma	[114,138]
AP1/ETS-COM-AP1 (~1900 bp)	AP1	TPA	NF-κB	hepatocytes	[126]
AP1 (4.1 kb)	Fra1 JUN JUNB JUND	basal conditions		aggressive breast cancer	[95]
AP1/ETS (~5300 bp)	JUN JUND	basal conditions	ETS	mouse transformed fibroblasts	[79,94]
AP1/ETS (~5300 bp)	↑ FOS	FGF2 → PKC → ERK JNK	cooperation of two AP1 sites, ETS	mouse transformed fibroblasts	[79,94]
AP1/ETS	JUND JUN JUNB	oncogenic ETS activation by JUN inhibition by JUND	oncogenic ETS	prostate cell line	[28]
AP1/ETS	JUND JUN JUNB JUN > JUND	low ERK	basal conditions, low ERK	prostate cell line	[28]
AP1/ETS	JUND JUN JUNB pJUND > JUN	ERK	ERK signaling cooperation with ETS	prostate cell line	[28]
AP1/ETS	AP1	IGF1 → ERK, PI3K	ETS	breast cancer cells	[119]
AP1	AP1	SDF → CXCR4 → p38PI3K	AP1 + Sp1 signaling	colon cancer cells	[110]
AP1/ETS	AP1	β-catenin-TCF4	β-catenin ETS	colorectal tumors	[85]
AP1	JUN	TGFβ-JNK		transformed keratinocytes	[128]
proximal and distal AP1	AP1	cAMP, retinoic acid	cis elements	mouse mammary carcinoma cells	[126]
AP1	AP1, NFκB	TGFβ → Src-NFκB Src-MAPK	dominant NFκB	ovarian cancer	[131]
CRE (−3.4 kb)	CREB	LFB3/HNF1B	crosstalk between cAMP and AP1 signaling	kidney cells	[125]

TPA: 12-O-Tetradecanoylphorbol-13-acetate; PKC: protein kinase C; CRE: cAMP response element; position of the promoter binding sites is shown in parentheses. ↑ increased binding to the promoter ↓ decreased binding to the promoter.

**Table 2:** Regulation of the AP1 site in the *PAI1* promoter.

Promoter Site	Transcription Factor	Pathway	Interfering Pathways	Cell Type	References
AP1 (~−79–50 bp)	FOS JUN	basal condition TPA cytokines	STAT NFκB	astrocytes	[175,176,177]
AP1 (~−79–50 bp)	FOS/JUND	fibrin proteolysis		fibroblast	[180]
AP1 (~−79–50 bp)	FOS JUN	thymosin β4 → ERK, JNK		endothelial cells	[183]
AP1 (~−79–50 bp)	FOS JUN	oxydative stress JNK	insulin pathways	pituitary cells	[207]
AP1 (~−79–50 bp)	JUN FOS	angiotensin II → MEK1,2 angiotensin II, high glucose—PKC	Sp1	vascular smooth muscle cells	[209,212]
AP1	JUN FOS	deprivation of aminoacids	cooperation with Sp1	melanoma cells	[210]
AP1	JUN	JNK		eyelid development	[211]
AP1	ATF2/JUN	lipoteichoic acid		mesothelial cells	[213]
AP1	JUN	thrombin-JNK		kidney cells	[178]
AP1	Fra1, Fra2, FOS	overexpression		breast cancer	[184]
AP1 (~−740–703 bp)	AP1	TGFβ	Smad alone or in cooperation with AP1	hepatoma cells	[187]
AP1 (~−740–703 bp)	JUN JUN/FOS JUND	placenta growth factor → JNK, HIFα, NADPH oxidase	HRE binding sites	endothelial cells	[214]
AP1	JUN, JUNB, FOS, Fra1	TGFβ	Smad3, Smad4, Smad2	breast cancer cells	[189]
AP1	AP1	TGFβ → ERK	Smad3/4	mouse fibroblasts	[174]
AP1	ATF2	TGFβ → JNK		colorectal cancer	[194]
AP1	AP1	TGFβ + EGF → p38	Smad	hepatocarcinoma	[195]
AP1	JUN	TGFβ + IL1β → ERK, Smad3	Smad	primary mesothelial cells	[196]
AP1	FOS	TGFβ + serum		renal epithelial cells	[149]
AP1	JUN ↓	basal and TGFβ induced KLF2 upregulation	Smad2	endothelial cells	[197]
AP1	JUN, FOS	fibrosis → JNK, ERK, p38, TGFβ		fibroblasts, kidney epithelial cells	[198]
AP1	AP1	high glucose, MEK, PKC glucose + TGFβ		vascular smooth muscle cells	[203,204]
AP1	AP1	oxidative stress		airway epithelial cells	[205]
AP1	AP1 ↓	actin cytoskeleton disruption → ERK, JNK ↓		mesangial cells	[181]
AP1	AP1	cytokine-mediated disruption—Src ERK ↑		kidney cells	[182]
AP1	JUN	silica treatment ERK		lung epithelial cells	[206]

TPA: 12-O-Tetradecanoylphorbol-13-acetate; PKC: protein kinase C; position of the promoter binding sites is shown in parentheses. ↑ increased binding to the promoter ↓ decreased binding to the promoter.

**Table 3:** Regulation of the AP1 sites in the *uPAR* promoter.

Promoter Site	Transcription Factor	Pathway	Interfering Pathways	Cell Type	References
AP1 (−70 bp)	FOS, JUN	basal conditions	Sp1 (Sp3) AP2	colon cancer	[217,232]
AP1 (−184 bp)	JUN, JUND, Fra1, FOS	basal conditions, TPA	NFκB Sp1 (Sp3) AP2 (TPA)	colon cancer breast cancer	[112,178,224,230,236]
AP1	JUN	JNK	Rac1 MEKK1	ovarian cancer cells	[224,230]
AP1 (−70 bp)	ATF2	RalA, Ras		human embryonic kidney cells colon carcinoma	[224,232]
AP1 (−184 bp)	JUN	RalA → Src, Ras		human embryonic kidney cells colon carcinoma	[224,232]
AP1 (−70 bp)	FOS JUN	NGF		pheochromocytoma cells	[233]
AP1 (−184 bp)	JUN JUND, Fra1	Src → JNK		colorectal cancer cells	[220]
AP1 (−184 bp)	JUND	TGFβ → MKK4, JNK		intestinal epithelial cells	[234]
AP1 (−184 bp)	AP1	basal conditions	Sp1 and AP2 sites	colorectal cancers	[235]
AP1	AP1	prostaglandin E2 → Src-EGFR-JNK, ERK, p38	NFκB	gastric cells	[236]
AP1	AP1	Macrophage-stimulating protein			[239]
AP1 AP1 (−70 bp)	JUN, Fra1	Wnt → β-catenin	β-catenin β-catenin + NFκB and Sp1	colon carcinoma cells	[240,241]
AP1 (−184 bp)	JUN, Fra1	acetylsalicylic acid		colon carcinoma cells	[242]
AP1	AP1	cadmium, hypoxia → MAPK		gastric cancer cells	[243]
AP1 (−184 bp)	AP1	UV B		keratinocyte cell line	[244]

TPA: 12-O-Tetradecanoylphorbol-13-acetate; PKC: protein kinase C; HRE: hypoxia response element; CRE: cAMP response element; position of the promoter binding sites is shown in parentheses.

## Data Availability

The original contributions presented in this study are included in the article. Further inquiries can be directed to the corresponding author.
